# Individual and Combined Inhalational Sedative Effects in Mice of Low Molecular Weight Aromatic Compounds Found in Agarwood Aroma

**DOI:** 10.3390/molecules26051320

**Published:** 2021-03-02

**Authors:** Kimberly P. Castro, Michiho Ito

**Affiliations:** Department of Pharmacognosy, Graduate School of Pharmaceutical Sciences, Kyoto University, 46-29 Yoshida-Shimoadachi-cho, Sakyo-ku, Kyoto 606-8501, Japan; castro.kimberly.77r@st.kyoto-u.ac.jp

**Keywords:** aromatic, agarwood, diethylene glycol, monoethyl ether, interaction, open field test, sedative

## Abstract

Agarwood is known to have a sedative effect and the less studied volatile aromatic constituents it contains may have contribution to the activity. In this study, two *Kyara* grade (highest-grade agarwood in Japan) samples were extracted using headspace-solid phase microextraction (HS-SPME) and analyzed through gas chromatography-mass spectrometry (GC-MS). Six low molecular weight aromatic compounds (LACs) and one structurally simple compound (diethylene glycol monoethyl ether) present in the aromas were individually evaluated for inhalational sedative activity in mice through open field test. Doses of 0.0001 g/L to 1 g/L were prepared for each compound and administered to mice (*n* = 6/dose/compound). Results revealed all compounds decreased spontaneous motor activity at almost all doses. Strongest sedative activity of each compound reduced total spontaneous motor activity by more than half against control, demonstrating their contribution to agarwood aroma and potential as independent sedating agents. Mixtures of compounds using their most effective dose were made and evaluated again for inhalational sedative effect. Interestingly, the combination of all compounds showed no significant effect and even caused stimulation in mice movements. This result suggests antagonistic-like interaction between the compounds, which is probably due to structural similarities. Consequently, it implies the other constituents present in agarwood, along with LACs, are also important to the overall sedative activity.

## 1. Introduction

The use of agarwood transcends spiritual, cultural, and regional differences as evidenced in ancient texts and scriptures: Romans burned it to honor the dead and the Gods, Greeks and Chinese included it in their pharmacopoeias, while Arabs and Indians incorporated it in perfumery being “one of the finest aromatics” [1,2]. Logic behind these beliefs and practices is as mystifying as to how agarwood is formed. The most plausible explanation goes that the resinous product is only produced when the heartwood of an *Aquilaria* tree (among other genera of family Thymelaeaceae) gets damaged by natural phenomenon or wounded by artificial means, with subsequent fungi infection [1,3,4]. Like a defense mechanism, agarwood production is the response of the tree to stress. Ironically, the same stress-induced resin is also known to have a calming effect [2,4,5,6]. This claim is supported by few modern pharmacological studies. Administration of either oil or extracts through oral, inhalational, or intraperitoneal injection demonstrated sedative activity as indicated by a decrease in spontaneous motor activity, hypothermic effect, and prolongation of hexobarbiturate-induced sleeping time in mice [2,5,7]. Likewise, individual constituents namely jinkoh-eremol, agarospirol, α-gurjunene, (+)-calarene, and benzylacetone resulted in sedation [7,8]. This makes agarwood a very promising starting material for finding novel or other existing compounds that may have activity. Concurrently, it is beneficial in trade and industry since strong competition in acquiring agarwood for medicinal purposes (e.g., for aromatherapy) will be minimized.

The high economic value of agarwood is something not to be taken lightly. To assess its worth, consuming countries like United Arab Emirates, Indonesia, and Malaysia have their own grading systems based on physical properties and geographical source [1,3,4,6,9,10,11]. In Japan, the highest grade is called *Kyara* (also known as *Kanankoh* or *Kynam*) which is mainly sourced from Vietnam [10,11]. The term originated from old Sanskrit *kara* meaning “black,” in reference to the desirable appearance of the resin-impregnated wood [10]. Additionally, *Kyara* is prized for the supreme quality of its scent, revered through dedicated incense rituals (kõdõ) [1,7,11]. The characteristic aroma is often likened to nobility and even used to express admiration of female beauty [1,10]. This inspired numerous studies to profile the aroma, but mostly to establish standardized criteria for identification and grading.

Constituents responsible for the characteristic aroma are generally grouped into sesquiterpenes, chromones, and other volatile aromatic compounds [3,4]. Majority of the composition are attributed to the first two [4]; thus, not much attention is given to the last group. However, most of the analyses were conducted on oil and extracts which may already lost some fragrant components due to rigorous extraction processes [12]. Agarwood is obviously best experienced through burning and direct inhalation of the heated wood, like the way it is traditionally appreciated in incense ceremonies. The same can be said when profiling the aroma. Hence, capturing it in its closest natural form is critical. One of the first few attempts in analyzing agarwood aroma directly from the wood found the composition was remarkably different from the solvent extract [13]. Several volatile aromatic compounds were also detected, which are believed to be pyrolysis products of other constituents [4,13,14]. A more recent study utilized a headspace (HS) preheating system and found as much as one-third of the incense smoke was made up of aromatics [9]. These compounds have a great contribution to agarwood aroma and probably play a role in its sedative activity as well. They may even have effects on their own, as seen in benzylacetone [7], thereby making them worth exploring. Moreover, the characteristic aroma of agarwood is often described as a product of the perfect harmony brought about by many compounds [4,14] and in this entirety the aroma is experienced. Hence, it is also important to investigate their possible interaction in terms of pharmacologic activity.

In this study, low molecular weight aromatic compounds (LACs) identified in the aroma of two high-quality agarwood (*Kyara* grade)—sampled by means of headspace-solid phase microextraction (HS-SPME) and analyzed through gas chromatography-tandem with mass spectrometry (GC-MS)—were individually evaluated for inhalational sedative activity in mice through open field test (OFT). Furthermore, mixtures of LACs (representing the components in each agarwood sample) using their most effective dose determined from the individual administration were evaluated to confirm sedative activity and to elucidate the interaction between compounds.

## 2. Results and Discussion

Two agarwood samples, herein denoted as K1 and K2, were used to determine LACs present in the aroma. The samples came in chips, which is the most traded form [3]. Based on physical properties and geographical source, both were classified as *Kyara*. K1 have a dark walnut to almost black surface and an inner creamy white portion upon incision. K2, on the other hand, have a brown mahogany shade all throughout. In terms of scent, K1 and K2 equally have the inexplicable characteristic agarwood fragrance that lingers; and their difference is practically indiscernible at room temperature. Distinction between the two became apparent when scraps of each sample were heated using an electric censer. K1 liberated a perplexing and heavier (caramel-like) aroma, whereas K2 released a clean, sweet, and refreshing fragrance.

### 2.1. Chemical Constituents of Agarwood Aroma Using HS-SPME GC-MS Analysis

HS-SPME works by virtue of multiphase equilibration system between the sample matrix, the gas phase surrounding the sample (i.e., headspace), and the fiber coating that extracts the analytes. Thus, solid samples like agarwood can be extracted without the need for any solvent or treatment. Moreover, the technique usually employs heating to facilitate mass transfer between phases [6,12], which is more than favorable in the present study. Aside from the fact that the usually encountered form of agarwood is smoke, application of heat was also necessary because the target compounds (i.e., the LACs) are presumed to be pyrolysis products of other constituents, particularly of the lignin and non-volatile chromones [4,13,14]. Indeed, the major chromone derivative agarotetrol was confirmed to break down into LACs, namely benzylacetone and benzaldehyde, upon heating [15]. A recent finding reported the number of identified compounds did not change significantly when agarwood was heated from 100 °C to 180 °C [11]. Therefore, samples in this study were heated at about 120 °C for 30 min before SPME. Subsequent analysis with GC-MS allowed separation of the extracted analytes as well as characterization of individual constituents. Results revealed chromatographic profiles of the two agarwood samples were different (Figure S1). Twenty-seven compounds were identified in the aroma of K1 (Table S1) representing 98.13% of the total volatiles, with more than half of the composition being spathulenol (51.78%). Meanwhile, a total of 35 compounds were detected in the aroma of K2 (Table S1) representing 99.28% of the total volatiles. Major components were δ- and α-guaiene (18.87% and 16.05%, respectively), along with capsidiol (14.73%). Nonetheless, the aroma of the two samples had 16 compounds in common and both were predominantly made up of sesquiterpenes. These profiles complemented previous analyses on high-quality agarwood smoke, which was characterized by the presence of more complex constituents (i.e., sesquiterpenes) and less aromatic compounds or pyrolysis products [8,13].

**Figure 1:**
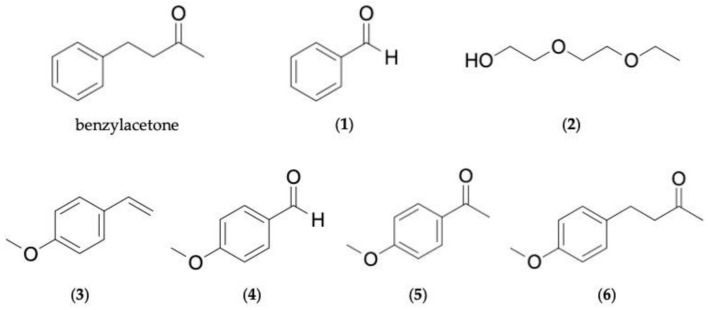
Chemical structures of compounds investigated for sedative effects in the study: benzylacetone (positive control); benzaldehyde (**1**); diethylene glycol monoethyl ether (DEGEE) (**2**); *p*-vinylanisole (**3**); *p*-anisaldehyde (**4**); acetanisole (**5**); and anisylacetone (**6**). All seven compounds were found in aroma of K1. Only benzylacetone, compounds **1**, **2**, and **4** were present in aroma of K2. (Compounds are numbered in order of elution from DB-WAX column.).

For clarity, LAC is defined in this study as a simple aromatic compound following rules of aromaticity. Having said that, six compounds out of the 27 identified in the aroma of K1 were classified as LAC; whereas only three LACs were present in the aroma of K2, namely: benzylacetone, benzaldehyde—**1**, and *p*-anisaldehyde—**4** (Figure 1). In addition to those three, the other LACs present in K1 were *p*-vinylanisole—**3**, acetanisole—**5**, and anisylacetone—**6**. All of them had been previously identified from analytical studies on either agarwood oil or smoke [4]. However, compound **5** was dismissed as an artifact by Hashimoto’s team when they analyzed agarwood that undergone neutralization treatment [16]. The structurally related acetophenone appeared more frequently [13,15]. Regardless, compound **5** was the exact compound identified in the present analysis; thus, it was the one tested in pharmacologic experiments. In addition to the abovementioned LACs, another compound was also included in the evaluation of sedative effects: diethylene glycol monoethyl ether (DEGEE)—**2**. It is evident DEGEE does not satisfy requirements for aromaticity, but the compound was deemed structurally simple enough and it also has a subtle, ethereal odor. Moreover, it was detected in both K1 and K2 aroma; although to the best of our knowledge, it had not been identified in previous agarwood analyses.

### 2.2. Sedative Activity of Individual LACs and DEGEE

**Figure 2:**
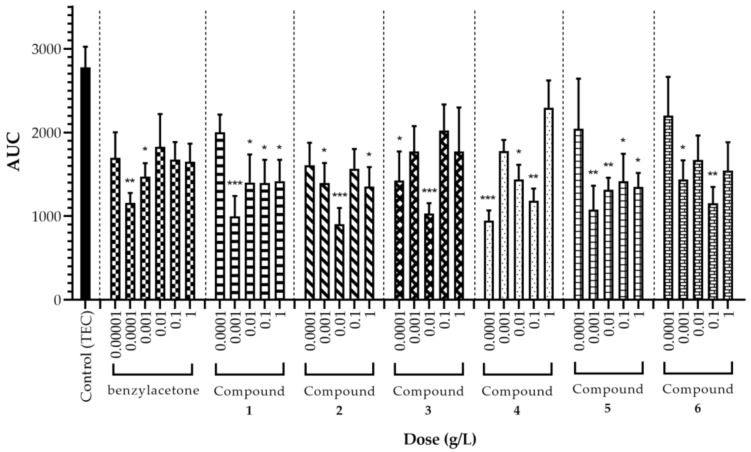
Total spontaneous motor activity of mice treated with the compounds shown in Figure 1. Data are expressed as mean ± SEM (*n* = 6). Statistical differences vs. control group were calculated using One-way ANOVA followed by Dunnett’s test. (* *p* < 0.05; ** *p* < 0.01; *** *p* < 0.001).

The sedative activity of each LACs and DEGEE were investigated through behavioral observation method, specifically the open field test (OFT). The simplicity of OFT and the straightforward interpretation of results makes it a suitable method in screening compounds for pharmacologic activity [17]. Reduced locomotion and prolonged immobility of mice in an hour-long OFT can be construed as sedation or sleep [17,18], which was also the indicator used by previous studies to evaluate sedative activity in mice [2,5,7,19,20,21,22,23,24,25,26]. With doses ranging from 0.0001 g/L to 1 g/L, a volume of 400 μL of pure compounds were individually administered to mice by vapor inhalation. These doses were determined based on the effective concentration range in earlier studies that employed the same experimental model [7,19,20,21,22,23,24]. Since the compounds under investigation have simple and related structures, one set of control group was considered appropriate for evaluation. Additionally, benzylacetone—an important volatile constituent in agarwood oil and smoke [7,13]—served a dual purpose of being a test compound and positive control. Benzylacetone had been established to possess an inhalational sedative effect through olfactory stimulation [7,19,20] and was used as a positive control in numerous animal studies [19,20,21,23]. The structure of benzylacetone is also closely related to the other compounds in the study; thus, evaluation of their sedative activity was more reliable. Coincidentally, benzylacetone is also abundantly emitted by some flowering plants like *Nicotiana attenuata* where it acts as the most attractant component [27]. Other plants found to have benzylacetone as a major component includes *Rhododendron anthopogonoides* and *Lavandula angustifolia* which are both used in traditional medicines to treat lung problems and to induce sleep, respectively [28,29,30]. At a dose of 0.0001 g/L, benzylacetone exhibited a strong sedative activity (58% reduction against control) confirming the validity of the experimental model. A similar dose-response pattern (i.e., U-shaped) shown in Figure 2 was also demonstrated from previous studies [7,19,21], although there were differences in the observed most effective dose maybe because of interpersonal variation. Hence, an additional 0.00001 g/L dose was performed to verify the results in the present study.

**Figure 3:**
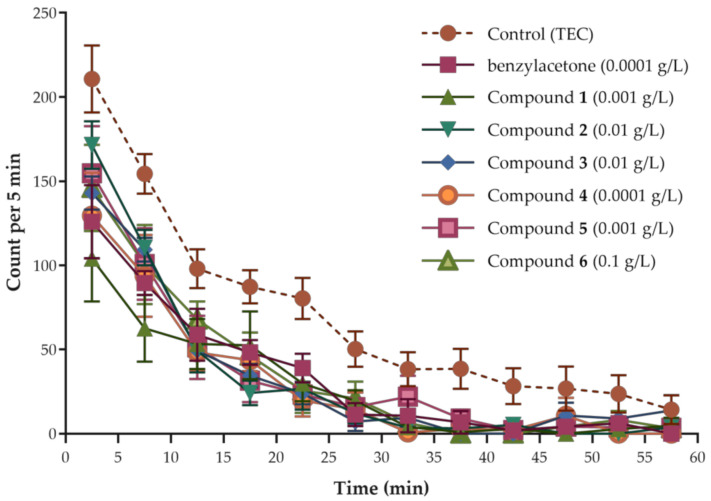
Locomotor transition activity of mice treated with the compounds investigated in the study at their respective most effective dose. Data are expressed as mean ± SEM (*n* = 6).

Inhalational administration results of the rest of the compounds are likewise presented in Figure 2. All of them reduced total spontaneous motor activity at almost all tested doses in a dose-dependent manner. This is a good indication that despite their low molecular weights, the compound molecules were successfully contained inside the make-shift open field arena; and thus, effectively administered to mice. The U-shape partly drawn by the area under the curve (AUC) graphs representing total spontaneous motor activity suggests a biphasic effect of the compounds; that is, sedative at lower doses and stimulatory at higher doses. This is a characteristic of drugs acting on the central nervous system (CNS) [31] and a description of CNS depressant effects brought by volatile aromatic hydrocarbons used as solvents, like the compounds investigated in this study [32]. The U-shaped pattern might not be too obvious because of the narrow therapeutic window and limited explored dose range. Moreover, a W-shaped pattern is apparent when analyzing the entire AUC graph of each compound (i.e., from 0.0001 g/L to 1 g/L), specifically compound **2**–**6**. It might be related to the presence of an additional substituent to the ring, particularly the alkoxy group which also happens to be in compound **2**. Nevertheless, reduced spontaneous motor activity at higher doses was dismissed as false sedative activity since excitation behaviors (e.g., jumping, excessive rearing) were seen in mice. This was true for all compounds. After the initial 20 min, the mice suddenly became indolent, yet generally remained awake and continuously moved until the end of experiments (Figure S2). Therefore, the effective dose of most compounds in this study appears narrow and more confined at the lower end of the explored range. The obtained results agree with the previous findings that individual administration of low doses of pure agarwood compounds is highly potent in terms of sedative activity [7]. Strongest sedative effect was observed at a dose of 0.001 g/L for compound **1** (benzaldehyde); 0.01 g/L for compound **2** (DEGEE); 0.01 g/L for compound **3** (*p*-vinylanisole); 0.0001 g/L for compound **4** (*p*-anisaldehyde); 0.001 g/L for compound **5** (acetanisole); and 0.1 g/L for compound **6** (anisylacetone) as evidenced by more than half reduction (64%, 67%, 63%, 66%, 61%, and 58%, respectively) in total spontaneous motor activity compared to control. Looking at the locomotor transition activity in Figure 3, a sharp decline in movements that almost approached zero in the first 30 min is observed in all compounds. This appears contradictory to the exploratory nature of mice. The less time spent in ambulation may be interpreted as easily reaching the comfortable state of sleepiness; and hence, forgoing the need to investigate the novel environment. Mice under the influence of LACs also exhibited low spontaneous motor activity at the outset of OFT; thus, suggesting an instantaneous and potent effect of these compounds.

Inhalation of compound **1**, a major constituent of *Tilia* (lime blossoms) essential oil used in folk medicine and aromatherapy for sedation, had already been reported to cause a decrease in spontaneous motor activity of mice [25]. Similar results were obtained by Miyoshi et al. wherein it was concluded that benzaldehyde (compound **1** in this study), as well as anisylacetone (compound **6** in this study) which is an analogue of benzylacetone, have potent sedative activity through the same route of administration [21]. These findings are reflected in the present study; thus, confirming the inhalational sedative activity of both compounds.

Compound **2** is a known solubilizer that can enhance skin and mucosal permeation of active ingredients in cosmetics and drug formulations [33]. However, its inertness as an excipient is an ongoing debate because it was reported to cause neurolepsis in mice (minimum symptomatic dose = 50 g/kg, intraperitoneal) and various CNS effects in rats (continuous inhalation at 0.27 and 4.5 ppm for 4 months) [33,34]. Results in this study further confirm that inhalation exposure of compound **2** at 0.01 g/L for an hour can cause sedation in mice.

Compound **3** is present in the essential oil of *Kaempferia galanga* which has a long history of fragrance use to improve sleep [26,35]. Recent pharmacological studies found inhalation of whole galangal oil and oral administration of its extract had a sedative effect in mice [22,26]. In the same study [22], it was inferred the sedative activity of galangal oil arises from the collective effect of the different components. Therefore, the decrease in spontaneous motor activity of mice caused by compound **3** suggests a possible contribution of the compound in the overall activity of galangal oil and indicative of its own sedative activity.

Compound **4**, an analogue of compound **1**, is a naturally occurring flavoring agent commonly found in spices such as *Pimpinella anisum* and *Foeniculum vulgare* [36]. It is interesting to note the two seemingly equal significant sedative effects (at 0.0001 g/L and 0.1 g/L) displayed in AUC graph of compound **4** (Figure 2). A similar motor activity pattern was demonstrated when thymol was evaluated for inhalational effect in mice; and through rotarod test, the decrease in spontaneous motor activity at higher dose was considered due to muscle relaxation instead of sedation [23]. Olfactory receptors are reported to exist in non-olfactory tissues like the skin and gut or intestine [37]. Hence, it is possible at 0.1 g/L dose, compound **4** exerts a direct effect on peripheral muscles through those receptors. Intraperitoneal administration of compound **4** was found to prolong pentobarbital-induced sleeping time in mice [36]. Additional investigation on this compound is required to further elucidate its effects.

Compound **5** is a structural analogue of acetophenone, which had been previously determined to be highly active in terms of inducing sedation in mice [21]. Therefore, resemblances in their structure might explain the similar sedative effect observed in compound **5**. Aside from agarwood, compound **5** is also a main volatile constituent of Propolis essential oil (collected from Albania), which is slowly becoming popular for its promising biological activities [38].

True to their name, the compounds under investigation in the study apart from compound **2** are well-known for their strong aroma. As such, it is obvious they are capable of stimulating olfaction by binding to olfactory receptors. The interaction initiates a signaling response that will eventually reach cortical and subcortical regions of the brain for higher processing [7,22,37,39]. Results obtained in the present study implies the outcome of that processing induced sedation in mice. It had been suggested before that the alternating π-bonds in a six-membered ring structure is necessary for the sedative effect [24]. The planar orientation and hydrophobic nature of the aromatic ring clearly enabled the LACs to interact easily to the flat, hydrophobic pocket of the olfactory receptor [40,41]. Moreover, the carbonyl group on the substituents probably provided additional linkage with the receptor through hydrogen bonding [40]; thus, resulting in a strong interaction. Correspondingly, the hydroxyl group of compound **2** seemed to allow interaction with the receptor in a similar manner (i.e., hydrogen bonding) [40]; thus, a sedative effect was seen despite the absence of aromatic ring. Future study using more structural analogues of the compounds is recommended to firmly establish structure-activity relationship.

### 2.3. Antagonistic-Like Interaction Between Test Compounds

**Figure 4:**
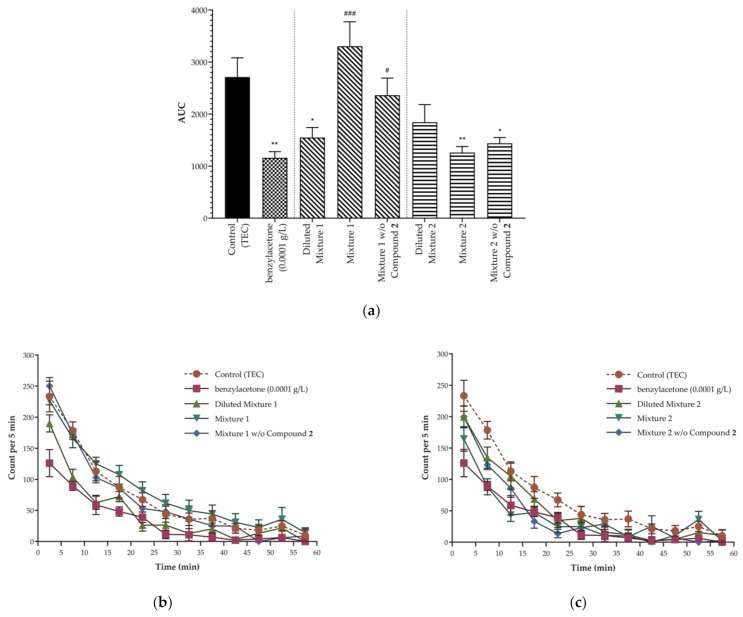
Total spontaneous motor activity (**a**), locomotor transition activity of mice treated with mixtures relating to K1 (**b**), and locomotor transition activity of mice treated with mixtures relating to K2 (**c**). Mixture 1, representing aroma constituents present in K1, contained all seven compounds; whereas Mixture 2, representing aroma constituents present in K2, only included benzylacetone, compounds **1**, **2**, and **4**. Data are expressed as mean ± SEM (*n* = 6). Statistical differences vs. control group and benzylacetone (0.0001 g/L) were calculated using One-way ANOVA followed by Dunnett’s test. (significant against control: * *p* < 0.05, ** *p* < 0.01; significant against benzylacetone: # *p* < 0.05, ### *p* < 0.001).

Compounds investigated in the study exhibit inhalational sedative effects individually. Given that they exist together to create the characteristic aroma of agarwood, it is important to see their combined effects or interaction from the pharmacologic perspective. There are several possible combinations of compounds in varying dose levels that can be tested. In order to simulate agarwood to some extent, mixtures of the compounds were made according to their presence in K1 and K2 aroma. The dose of each compound in the mixture was based on their respective most effective dose determined from individual administration. Mixture 1, representing LACs in K1 aroma, was composed of 1/7-part benzylacetone at 0.0001 g/L; 1/7-part compound **1** (benzaldehyde) at 0.001 g/L; 1/7-part compound **2** (DEGEE) at 0.01 g/L; 1/7-part compound **3** (*p*-vinylanisole) at 0.01 g/L; 1/7-part compound **4** (*p*-anisaldehyde) at 0.0001 g/L; 1/7-part compound **5** (acetanisole) at 0.001 g/L; and 1/7-part compound **6** (anisylacetone) at 0.1 g/L. On the other hand, Mixture 2, representing LACs in K2 aroma, was composed of 1/4-part benzylacetone at 0.0001 g/L; 1/4-part compound **1** (benzaldehyde) at 0.001 g/L; 1/4-part compound **2** (DEGEE) at 0.01 g/L; and 1/4-part compound **4** (*p*-anisaldehyde) at 0.0001 g/L. Since compound **2** is structurally different and had not been identified from agarwood analyses before, separate mixtures were also created without it (i.e., Mixture 1 without Compound **2** and Mixture 2 without Compound **2**). Initially, four different mixtures were made. Each mixture (400 μL) was administered to mice and the sedative effects were evaluated using the same experimental model employed during individual administration. Results are shown in Figure 4.

Interestingly, mice treated with Mixture 1 showed an increase in spontaneous motor activity by 20% compared to the control group. Excitation behaviors were also observed such as jumping, unsupported rearing, and seemingly attempting to climb walls. Moreover, it is evident from the locomotor transition activity chart (Figure 4b) the mice kept moving during the entire OFT. This suggests possible antagonism between the seven compounds. The reason may be due to the large number of different molecules (i.e., seven different compounds) present in the mixture which crowded and overwhelmed the receptors; thus, stimulated movements of mice. In contrast, mice treated with Mixture 2 exhibited 54% reduction in spontaneous motor activity against control, indicating the combination of benzylacetone, compounds **1**, **2**, and **4** has significant sedative activity. This is probably because of the smaller number of different molecules (i.e., only four compounds) present in the mixture. Although the sedative activity of Mixture 2 and single administration of benzylacetone (0.0001 g/L) were not significantly different, it appears the latter is more effective in inducing sedation in mice. Hence, further supporting the influence of the number of diversified molecules in the sedative activity.

The absence of compound **2** in Mixture 1 decreased spontaneous motor activity by 13%, but hardly enough to generate a significant effect. Removing compound **2**, which has a mucosal permeation enhancing functionality [33], likely decreased contact time of the LACs in the mucus layer of mice. Accordingly, this relieved overcrowding of the large number of molecules rushing to the receptor; and a decrease in spontaneous motor activity was observed when compound **2** was removed from the mixture. On the other hand, a 47% reduction in spontaneous motor activity was observed when compound **2** was removed from Mixture 2, which was slightly less effective than having it in the mixture. This result might appear contradicting with the preceding argument but to reiterate, a relatively small number of molecules was present in Mixture 2. Therefore, further removal of compound **2** probably means decreased contact time of the LACs in the mucus layer and less chances for the few molecules present in interacting with the receptors. In addition, aldehydes (i.e., compounds **1** and **4**) which made up most of Mixture 2 are prone to fast enzymatic conversion in mouse mucus [42]. Thus, it is possible the lack of permeation enhancement brought by compound **2** placed the LACs in Mixture 2 at a disadvantage, resulting in less effective sedation in mice. Additional experiments on compound **2** with other combinations of compounds or even with a single compound may aid in better demonstration of its effects.

To further confirm the interaction between compounds, Mixture 1 and 2 were each diluted to 1/10 of the original dose mixture. Results are incorporated in Figure 4. Diluted Mixture 1 significantly reduced spontaneous motor activity of mice, which is almost half of the original mixture. This shows a lower dose of Mixture 1 caused a significant sedative effect in mice, supporting the results obtained using the original mixtures; that is, fewer molecules have less tendency to overcrowd receptors and oppose each other’s sedative activity. Likewise, loss of sedative activity of Diluted Mixture 2 corroborates the assumption made on the influence of the number of molecules present in the mixture, as well as the less effective sedative effect observed when compound **2** was removed from the original mixture. There were already a small number of molecules in Mixture 2, enough to generate a sedative effect; thus, a further decrease in molecule count (e.g., through dilution or removal of compound **2**) results in less opportunity for interaction with receptors and loss of sedative activity.

The exact nature of the observed interaction is difficult to ascertain and it is presumptuous to outright claim antagonism between test compounds. Nonetheless, it is possible the many different compound molecules were competing for the same target receptor. Resemblances between compounds investigated in the study are quite evident in Figure 1. Structurally related aromatic compounds—specifically phenyl ethers, aromatic ketones, anisole and derivatives—were reported to activate the same dorsal region of the mouse olfactory bulb [37], attesting the likelihood of competition between the test compounds. The presence of more LACs (i.e., Mixture 1) likely created a tougher competition between structurally alike compounds, while the presence of fewer LACs (i.e., Mixture 2) means less competition; thus, a more effective sedative effect was seen in the latter. Target competition between structurally related compounds was also the assumption drawn by Suberu et al. when anti-malarial active artemisinin was combined with its analogues 9-epi-artemisinin and artemisitine having anti-plasmodial activity of their own but still ended up antagonizing artemisinin’s activity [43].

Furthermore, aromatic compounds (or odorants, in general) are known to be capable of binding with several olfactory receptors; and conversely, olfactory receptors can accommodate several aromatic compounds. Still, activation of the receptor is not only dependent on the binding affinity of the aromatic compound with the receptor, but also on the intrinsic activity of the aromatic compound or the ability to activate the receptor [39]. Taking Mixture 1 as an example, the higher dose of compound **6** (0.1 g/L) relative with other compounds present suggests it had the largest number of molecules in the mixture, which implies higher chances of binding with the receptor. However, this also means compound **6** is weakly activating since it required that much dose to elicit a sedative effect or a 58% reduction in spontaneous motor activity. Compound **6** possibly occupied but only partially activated the receptors, leaving the strongly activating molecules of other compounds incapable of binding with the receptors. As a result, no significant effects or only comparable sedative activity with a single administration of benzylacetone were observed using the mixtures. Therefore, similarities in the structure of compounds and the number of molecules in a mixture may justify the demonstrated antagonistic-like interaction between LACs and the resulting decrease or loss of sedative activity.

The sedative activity of agarwood oil administered via inhalation and intraperitoneal injection had been successfully demonstrated from previous pharmacological studies [2,7]. In both cases, majority of the oil composition were sesquiterpenes. Takemoto et al. specifically identified α-gurjunene and (+)-calarene as the main volatile constituent in the agarwood oil and confirmed the inhalational sedative effect of individual administration of pure compounds [7]. A lower dose of the individual sesquiterpenes as compared to their actual content in the oil was also found to be more effective in exerting the sedative effect. Clearly, sesquiterpenes present in agarwood oil has sedative effects. In the same study, the effect of pure (+)-calarene was compared to that of (+)-calarene-containing crude extract of spikenard. It was revealed the crude extract has a wider range of effective doses, implying the many compounds present in the extract caused a collective sedative effect through a combination of various biological activities [7]. Relating it to the present study, the LACs have sedative effects, and along with other constituents (e.g., sesquiterpenes)—which are probably acting on different receptors, through different mechanisms or signaling response—they give rise to the overall sedative activity of agarwood.

## 3. Materials and Methods

### 3.1. Chemicals, Reagents, and Samples

Triethyl citrate (TEC) (purity > 98%, Merck, Kenilworth, NJ, USA), an odorless solvent, was used as a vehicle for the test compounds. Benzylacetone (purity > 95%, Tokyo Chemical Industry, Co., Ltd., Tokyo, Japan) is known to have a sedating effect [7,19,20,21,23], thus it also served as a positive control. Benzaldehyde (purity ≥ 98%), diethylene glycol monoethyl ether (purity ≥ 98%), *p*-anisaldehyde (purity ≥ 99%), and acetanisole (purity ≥ 95%) were purchased from Nacalai Tesque, Inc (Kyoto, Japan). Anisylacetone (purity > 96%) was bought from Tokyo Chemical Industry, Co., Ltd. (Tokyo, Japan). *p*-vinylanisol (purity ≥ 97%) was purchased from FUJIFILM Wako Chemical Co., Ltd. (Osaka, Japan). All chemicals used were of the highest grade available.

High-quality agarwood chip samples were provided by one of the long-established incense shops in Kyoto, Japan. Both wood chips were neatly cut (approximately 2 cm long, 0.5 cm wide) having smooth surfaces. Pleasant smells were also easily perceptible.

### 3.2. Agarwood Analysis Using HS-SPME GC-MS

Aroma of agarwood samples were extracted using HS-SPME and analyzed through GC-MS. One milligram of agarwood chip sample, shredded by hand, was placed in an SPME vial (Supelco, Inc., Bellefonte, PA, USA). The vial was heated on a hot plate for 30 min. The temperature of the bottom surface of the vial was continuously measured using a digital thermometer and strictly monitored by adjusting the controls of the hot plate. The actual heating temperature ranged from 126 °C to 148 °C. After heating, the SPME fiber (polydimethylsiloxane, 100 μm; Supelco, Inc., Bellefonte, PA, USA) was inserted into the headspace at room temperature for 10 min to adsorb the volatile components.

Qualitative analysis of the volatile components was performed using GC-MS (GC-2030/GCMS-QP2020 NX; Shimadzu Corporation, Kyoto, Japan) under the following operating conditions: column was DB-WAX (60 m × 0.25 mm, film thickness: 0.25 μm; Agilent Technologies, Santa Clara, CA, USA); column oven program was 60 °C initially held for 2 min, increased by 4 °C/min to 120 °C and maintained for 10 min, then increased by 3 °C/min until 210 °C, further increased by 5 °C/min to 230 °C, and maintained for 9 min. Injection temperature was 160 °C; carrier gas was helium set at 1 mL/min; and MS interface temperature was 240 °C. Peaks were identified based on retention times and mass fragmentation patterns available on the National Institute of Standards and Technology (NIST) database. Linear retention index values of volatile components were obtained by running a mixture of *n*-alkanes (C_10_–C_22_) as standard under the same condition. Relative amount of the compounds was estimated using peak area normalization method since using an internal or external standard is not feasible given the solid state of the samples.

### 3.3. Animal Experiments

#### 3.3.1. Animals

Four-week-old male ddY mice (weighing 18–20 g) were purchased from Japan SLC, Inc (Shizuoka, Japan). Upon arrival, the animals were housed in colony cages (33.8 cm wide, 22.5 cm long, 14 cm high; 6 mice per cage) lain with approximately 1 cm-thick chip wood bedding. Cages were kept in the animal housing facility at a temperature of 23 ± 2 °C and relative humidity of 50 ± 10%, under a 12 h light-dark cycle with the light phase beginning at 8:00. All mice had free access to tap water and standard pellet chow. They were allowed to acclimatize for a week prior to use in experiments.

Behavioral observations were performed between 9:00 and 17:00 simulating identical temperature and humidity conditions as stated. Animals were brought to the standard-lit testing room in their home cages an hour before they were subjected to OFT. Only naïve mice were used for each testing.

#### 3.3.2. Evaluation of Spontaneous Motor Activity through OFT

The experiment was carried out as described in previous studies [7,19,21,22,23,24]. Benzylacetone and each test compound was dissolved in TEC to prepare doses of 0.0001, 0.001, 0.01, 0.1, and 1 g/L. The open field arena (60 cm wide, 30 cm long, 34 cm high) was made of clear acrylic, with floors (divided into 10 cm× 10 cm squares) and walls in white background. Two filter paper discs (90 mm diameter) were cut in half and attached using adhesive tape to each top corner of the arena. Then, each piece was loaded with 100 μL of the prepared dose of either benzylacetone (positive control), test compound or mixture (400 μL in total for every administration). The arena was immediately covered allowing the fragrant molecules to permeate inside by natural diffusion. After exactly 1 h, a mouse was placed in the center; and ambulation was recorded by a video camera suspended above the arena for another hour. Spontaneous motor activity was evaluated by counting the number of squares crossed with the four paws every 5 min. AUC, representing total locomotor activity, was calculated using the trapezoidal rule. The same procedure was followed for the control group, replacing the test compound with a similar volume of TEC only.

After each experiment, the arena was wiped with 70% ethanol and soft micro wiper, then left to dry for about 5 min to remove any olfactory cues from the previous mouse before proceeding to the next trial. Control (TEC) and treatment groups were randomly and simultaneously performed throughout the study to account for variation; and at the same time, to reduce the number of mice. The results were reproducible; thus, using six mice for each administration group were found to be sufficient.

### 3.4. Statistical Analysis

All values are expressed as mean ± standard error of the mean (SEM). Analyses were carried out using one-way analysis of variance (ANOVA) followed by Dunnett’s multiple comparison test. GraphPad Prism version 8 (GraphPad Software, San Diego, CA, USA) was used to perform all statistical analyses. A *p*-value < 0.05 was considered statistically significant.

## 4. Conclusions

The characteristic aroma of agarwood is made up of numerous complex constituents. Among them are LACs which had been previously identified as consistent pyrolysis products of non-volatile chromones. In the present study, these LACs were proven to have inhalational sedative effects, demonstrating their importance in the aroma as well as substantiating the use of agarwood as a sedative in traditional medicines. Similarly, this suggests the potential of LACs as independent sedating agents. The compounds may be investigated further using other animal models for better insight on the sedative effects.

Structural similarities of the LACs seem to cause antagonistic-like interaction between compounds, resulting in a decrease or loss of sedative effect. This means the overall sedative activity of agarwood arises from the collective effect of the different classes of constituents (i.e., LACs, sesquiterpenes) it contains. A future study exploring a broad range of doses and compound combinations is recommended to firmly establish interaction between constituents.

## Data Availability

The data presented in this study are available on reasonable request from the corresponding author.

## Supplementary Materials

The following are available online, Figure S1: GC-MS chromatograms of K1 and K2 aroma, Table S1: Chemical constituents and relative amounts identified in aroma of K1 and K2, Figure S2: Locomotor transition activity of mice treated with each compound at all tested doses.

## Sample Availability

Not available.

## Abbreviations

LAC	Low molecular weight aromatic compound
HS-SPME	Headspace-solid phase microextraction
GC-MS	Gas chromatography-mass spectrometry
OFT	Open field test
DEGEE	Diethylene glycol monoethyl ether
TEC	Triethyl citrate
AUC	Area under the curve
CNS	Central nervous system
