# Supplementary material for: Individual and Combined Inhalational Sedative Effects in Mice of Low Molecular Weight Aromatic Compounds Found in Agarwood Aroma
Source: Molecules. 2021 Mar 2;26(5):1320. doi: 10.3390/molecules26051320 (PMC7958121; doi:10.3390/molecules26051320)
Supplement: Supplementary file 1 [file molecules-26-01320-s001.pdf]

Supplementary Materials

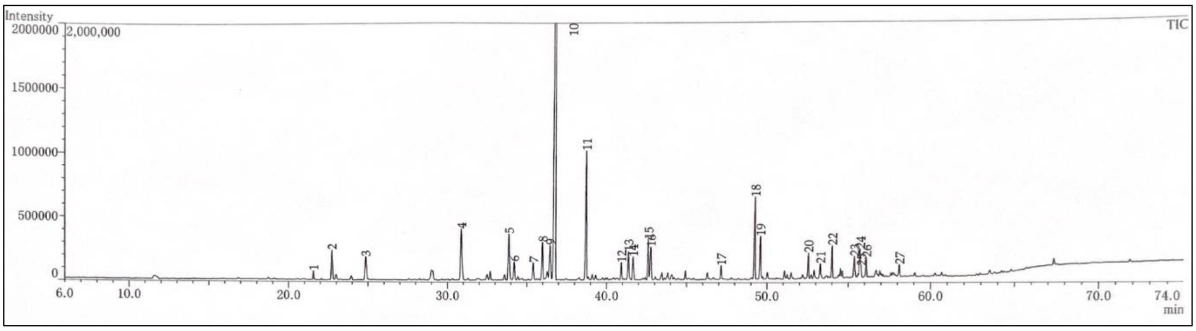

(**a**)

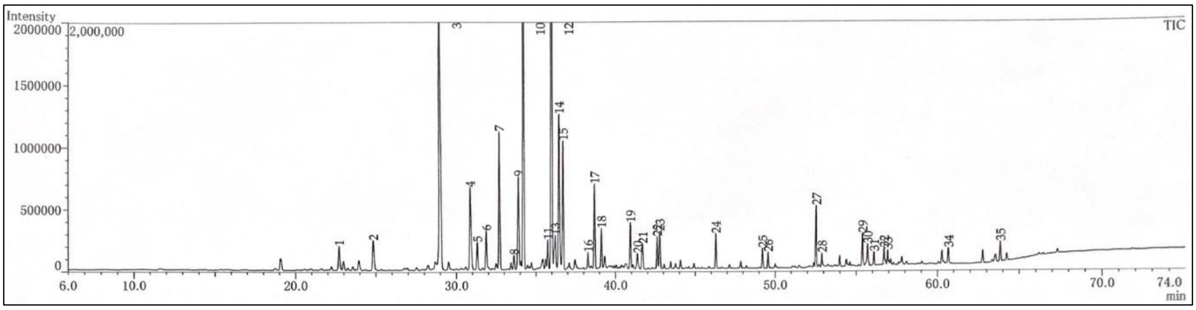

(**b**)

Figure S1. GC-MS chromatograms of K1 (**a**) and K2 (**b**) aroma. Peak no. 2 in K1 and Peak no. 1 in K2 were also found in blank.

Table S1. Chemical constituents and relative amounts identified in aroma of K1 and K2.

Compound	Linear Retention Index	Relative Amount (%) [a]	
		K1	K2
Octaethylcyclotetrasiloxane	1458	0.30	—
**Benzaldehyde**	1530	1.58	1.34
α-Guaiene	1617	—	16.05
**Diethylene glycol monoethyl ether**	1648	3.91	4.10
9-(1-Methylethylidene)-1,5-cycloundecadiene	1656	—	0.98
8,8-dimethyl-9-methylene-1,5-cycloundecadiene	1665	—	1.44
Isosativene	1680	—	5.42
1,5,9,9-Tetramethyl-1,4,7-cycloundecatriene	1695	—	0.32
***p*-vinylanisole**	1699	3.23	—
Selina-4,11-diene	1700	—	3.90
Capsidiol	1705	0.95	14.73
(−)-Borneol	1726	0.85	—
Eremophilene	1732	—	0.95
δ-Guaiene	1736	2.25	18.87
β-Selinene	1740	—	1.90
α-Selinene	1744	2.51	6.41
Spathulenol	1749	51.78	4.99
1(10),11-Eremophiladien-9-ol [b]	1775	—	0.44
α-Curcumene	1782	7.57	3.12
(−)-Nootkatene	1789	—	1.27
Copaene	1829	0.82	1.46
1-Hydroxymethyl-5,8,9-endo-10-exo-tetramethyltricyclo[6.3.0.0(5,11)]undecane	1840	1.46	0.31
3-Carene	1847	1.46	—
Isovalencenyl formate	1848	—	1.07
**Benzylacetone**	1870	2.15	0.98
1(10),11-Eremophiladien-9-ol [b]	1874	2.21	1.45
9H-Cycloisolongifolene, 8-oxo-	1959	—	1.08
Isolongifolene, 4,5,9,10-dehydro-	1980	0.59	—
***p*-anisaldehyde**	2037	4.53	0.56
Liguloxide	2047	2.28	0.44
Palustrol	2133	1.19	1.79
α-Santal	2144	—	0.32
**Acetanisole**	2154	0.57	—
γ-Gurjunenepoxide-(1)	2175	1.58	—
γ-Eudesmol	2215	1.14	—
Pogostol	2216	—	1.00
(2,4a,5,8a-tetramethyl-1,2,3,4,7,8-hexahydronaphthalen-1-yl) acetate	2225	1.54	0.62
(+)-Valerianol	2227	0.38	—
4-(1,5-Dihydroxy-2,6,6-trimethylcyclohex-2-enyl)but-3-en-2-one	2236	0.88	0.29
3,11-Eudesmadien-2-one	2254	—	0.36
(−)-α-Costol	2261	—	0.30
**Anisylacetone**	2295	0.42	—
2(1H)Naphthalenone, 3,5,6,7,8,8a-hexahydro-4,8a-dimethyl-6-(1-methylethenyl)-	2369	—	0.43
(+)-4,11-Eudesmadien-3-one	2642	—	0.59
Total		98.13	99.28

Constituents in bold text are the compounds investigated for sedative effects in the study.
Linear retention index relative to the retention time of *n*-alkane series (C_{10}–C_{22}) on DB-WAX column.
[a] percentage area of peaks in total ion chromatogram of GC-MS
[b] MS spectra were similar, and it was not possible to narrow down the peaks to one compound.
— absent

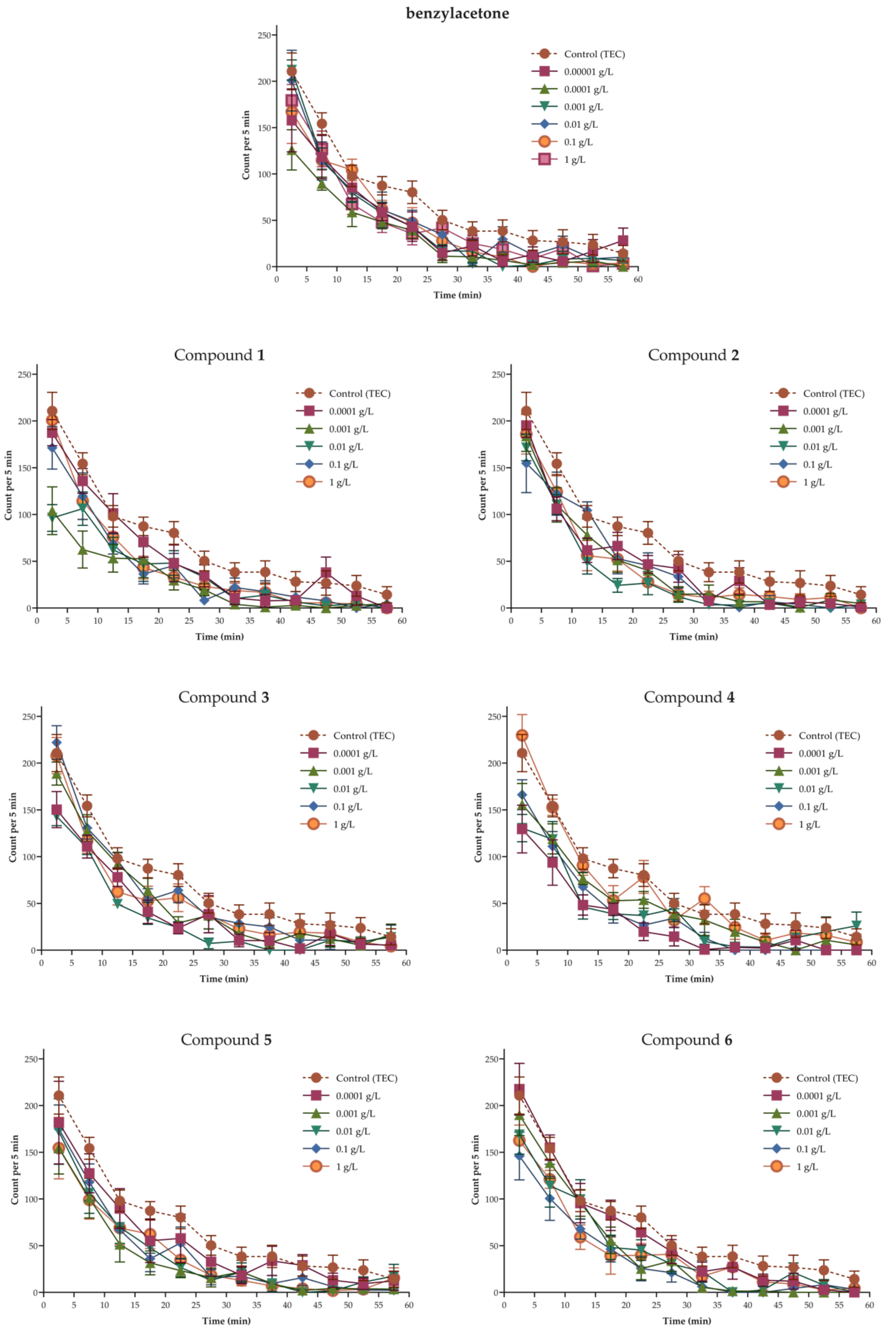

Figure S2. Locomotor transition activity of mice treated with each compound at all tested doses. Mice administered with higher doses continuously moved throughout time course. Data are expressed as mean ± SEM (n = 6).
